# Down Syndrome and Obesity Management: Lifestyle Observations Made in the Framework of the GO-DS21 Project and Clinical Recommendations

**DOI:** 10.3390/nu18060886

**Published:** 2026-03-11

**Authors:** Maria Gomis-González, Li F. Chan, Anne Hiance-Delahaye, Mara Dierssen, Andre Strydom, Rafael de la Torre, GO-DS21 Consortium

**Affiliations:** 1 Integrative Pharmacology and Systems Neurosciences Research Group, Neurosciences Research Program, Hospital del Mar Research Institute, 08003 Barcelona, Spain; mgomis@researchmar.net; 2 Centre for Endocrinology, William Harvey Research Institute, Faculty of Medicine and Dentistry, Queen Mary University of London, Charterhouse Square, London EC1M 6BQ, UK; l.chan@qmul.ac.uk; 3 Institut Jérôme Lejeune, 75015 Paris, France; anne.hiance-delahaye@institutlejeune.org; 4 Centre for Genomic Regulation (CRG), 08003 Barcelona, Spain; mara.dierssen@crg.eu; 5 The Barcelona Institute for Science and Technology (BIST) Centro de Investigación Biomédica en Red de Enfermedades Raras Instituto de Salud Carlos III, 28029 Madrid, Spain; 6 Department of Medicine and Life Sciences (MELIS), Universitat Pompeu Fabra, 08003 Barcelona, Spain; 7 Department of Forensic and Neurodevelopmental Sciences, Institute of Psychiatry, Psychology & Neuroscience, King’s College London, London SE5 8AB, UK; andrestrydom@kcl-ac.uk; 8 South London and the Maudsley Foundation NHS Trust, London SE5 8AZ, UK; 9 Centro de Investigación Biomédica en Red de Fisiopatología de la Obesidad y la Nutrición, Instituto de Salud Carlos III, 28029 Madrid, Spain

**Keywords:** Down syndrome, obesity, overweight, nutrition, physical activity, prevention

## Abstract

**Introduction/background:** Individuals with Down syndrome (DS) face a higher and earlier risk of overweight and/or obesity than the general population, influenced by factors like limited physical activity, suboptimal diet, stress, and sleep disorders. Despite the impact these lifestyle factors can have, there is a significant lack of robust, evidence-based guidelines for managing overweight and obesity in these individuals, particularly for the adult population. **Results:** Based on findings from an observational study across three European sites, within the EU Horizon-2020 GO-DS21 project, in this short communication, we highlight some key insights for developing effective prevention and management strategies for overweight and obesity in people with Down syndrome. **Conclusions:** In general terms, effective overweight and obesity management demands a holistic approach, integrating tailored diet and physical activity and actively addressing co-occurring health conditions. Moreover, the active participation of the family and peers is crucial for promoting sustainable healthy habits and for enhancing the overall quality of life of people with Down syndrome.

## 1. Introduction

Due to several environmental and lifestyle risk factors, including limited physical activity, poor dietary quality, stress, and some sleep disorders, individuals with Down syndrome (DS) are more likely to develop overweight and obesity than their neurotypical peers. Furthermore, those with DS have greater rates of overweight and obesity than those with other intellectual disabilities or other intellectual and developmental disabilities [1].

There is a crucial gap in the medical care of the DS population and a need for more research into the factors associated with obesity, with an absence of evidence on the management of obesity, particularly for adults [2,3]. In fact, the recent U.S. guidelines on managing obesity in DS are mostly aimed at children [4]. To address this gap, this brief communication aims to provide practical clinical recommendations for individuals with DS and their families to support weight management and obesity prevention; this brief communication highlights key issues for preventing and managing obesity. These recommendations are based on a synthesis of the current literature plus several studies performed in the framework of the EU Horizon 2020 program’s GO-DS21 (Gene overdosage and comorbidities during the early lifetime in DS) project. The first studies concerned the analysis of UK electronic health records [5,6]. One was focused on the analysis of the prevalence of comorbidities across the lifespan in subjects with Down syndrome. The second, more specifically focused on the prevalence of diabetes and obesity in the lifespan of subjects with Down syndrome. These findings obtained by analyzing health electronic records were combined with findings from an observational clinical study carried out in three European sites in a population with DS aged 12 to 45 [7]. The present report is a set of recommendations stemming from the studies performed within the GO-DS21 project combined with recommendations issued from other Down syndrome organizations. The final aim is to offer a practical framework for the prevention and treatment of overweight and obesity in the Down syndrome population.

## 2. Methods

The clinical guidelines presented here are based on evidence available in the existing literature, including previously published guidelines, together with the results obtained from a GO-DS21 project sub-study. These recommendations have been developed following a comprehensive analysis of the data generated within this research project, ensuring that they reflect the evidence gathered directly from the study population.

The study was conducted across three clinical sites with expertise in the field of DS including: the King’s College of London (KCL, London, UK), the Hospital del Mar Research Institute (HMRI, Barcelona, Spain) and the Institut Jérôme Lejeune (IJL, Paris, France). Patient recruitment took place between June 2020 and August 2024. All the information collected in the GO-DS21 study—upon which these guidelines are based—was obtained during a single study visit, ensuring consistency and standardization of data collection across centers.

A summary of the study’s population characteristics can be found in Table 1.

The primary data in the GO-DS21 project was collected and analyzed using standardized questionnaires and protocols. Specifically:

- Dietary intake was assessed using a Food Frequency Questionnaire (FFQ) specific to each country, allowing the evaluation of macro- and micronutrient intake and the quality of the diet. Specifically, we used: the French MetaCardis FFQ for France (159 items) [8], the EPIC-Norfolk FFQ for the UK (130 items) [9] and the PREDIMED FFQ for Spain (140 items) [10].
- IQ scores were measured through the administration of the Leiter-3, a non-verbal test administered by trained psychologists [11].
- Physical activity levels were quantified using the Spanish short version of the Minnesota Leisure Time Physical Activity Questionnaire (VREM) [12].
- Parental BMI was self-reported by parents during the interview conducted for data collection.

## 3. Results

### 3.1. Co-Occurring Conditions in People with DS

People with DS often present associated comorbidities. An analysis of the electronic health-record data from the UK Clinical Practice Research Datalink, as part of the GO-DS21 project, revealed that multimorbidity is significantly more common and develops earlier in individuals with DS than in the general population [5]. These individuals show a distinct pattern of multiple morbidities, with an age-related incidence, frequently involving endocrine, neurological, and cardiovascular issues. Some of the most found comorbidities are obesity, hypothyroidism, obstructive sleep apnea, or constipation, which may be co-occurring and inter-related and may be impacted by environmental and lifestyle factors. These findings underscore the complex, lifelong healthcare needs of people with DS, suggesting that a holistic approach is necessary to support health and prevent long-term conditions. Offering regular comprehensive health checks, starting early in life, may improve screening, prevention and treatment for people with DS, as well as improve their quality of life.

### 3.2. People with DS Are More Prone to Being Overweight/Obese

Our findings suggest that the prevalence of overweight/obesity in DS participants may be similar to that in the general population. However, DS subjects appear more likely to present obesity at an earlier age [4], with observations indicating that people with DS often display a higher body mass index (BMI) when younger than that in the control population [5]. Notably, while obesity is a recognized risk factor for type 2 diabetes mellitus in the general population, this association is particularly significant in the DS population, where its incidence is up to four times higher at younger ages than that in controls [6].

### 3.3. The BMI of People with DS Is Associated with the BMI of Their Parents

In the general population, a poor maternal pregnancy diet quality has been associated with a higher level of insulin resistance in early childhood [13], and both maternal and paternal overweight/obesity exhibited a similar link between their BMI and the behaviors expressed by their offspring [14]. In DS we observed an association with maternal BMI and marginally with paternal BMI [7]. These findings suggest that the family context, especially maternal health and habits, may play a critical role in obesity prevention and treatment in the DS population. Consequently, family-based dietary approaches could be considered during the early neurodevelopmental stages, whereas many existing DS weight management interventions currently focus primarily on unique individual metabolic and physical concerns.

### 3.4. Dietary Guidance Should Be Country-Specific Due to Varying Dietary Patterns and Quality

In a recent review of dietary patterns in subjects with DS, it was reported that they are often characterized by low consumption of vegetables, fruits, and dairy products, as well as high consumption of meat and sweet snacks [15]. While our findings in the GO-DS21 project suggest that the dietary quality of people with DS was comparable to that of the general population [7], this does not mean that dietary improvements are unnecessary in this population. Using the Healthy Eating Index-2020, we found that dietary quality of people with DS varied between European countries with scores generally falling below the recommended levels for a high-quality diet. Therefore, there may be potential benefits in implementing dietary interventions in individuals with DS to improve diet quality. These interventions should be based on effective diet programs tailored to the cultural and country context, considering socio-cultural factors, local food availability and traditions.

### 3.5. A Higher Standard of Nutrition Quality Could Be Linked to Better Cognitive Ability

Within the GO-DS21 study, in an exploratory study in a subset of subjects with cognitive data available, healthy diets (characterized by a predominance of high-quality proteins, abundant vegetable consumption, and whole grains) and a healthy lipid profile were associated with a higher intelligence quotient (IQ), whilst higher adiposity, excess weight and low-quality fat consumption were linked to lower IQ scores [7].

While these findings suggest a link, the association between a good-quality diet and a higher IQ is already known in other contexts. A good diet quality is critical during pregnancy, which is associated with higher full-scale IQ scores for adolescents, particularly in verbal comprehension and matrix reasoning [16]. Dietary patterns in early- and mid-childhood are associated with differences in brain morphology, which may partially explain the relationship between dietary patterns and neurodevelopment in children [17]. Although the nature of our cross-sectional data limits the ability to establish causality, these results support the hypothesis that the maximal effect of diet on IQ might occur in the early stages of neurodevelopment. Considering that DS is a neurodevelopmental disorder and that individuals with DS face greater cognitive challenges, dietary interventions could potentially be efficacious in this population, especially in the early lifespan stages. Thus, weight management in DS could be considered not only to be a metabolic health goal but also a potential cognitive health priority.

### 3.6. Supporting Exercise to Manage Weight

In the GO-DS21 study, we showed that lower levels of physical activity were negatively correlated with age and positively correlated with greater BMI, indicating that mid-life participants engaged in less exercise [7]. People with DS are more likely than the general population to exhibit sedentary behaviors, with low levels of physical fitness, and limited opportunities for physical exercise. In people with DS, exercise may be particularly important not only for preventing obesity but also for preserving cardiovascular health, bone health, and muscle strength, since loss of bone density and muscle begin at earlier ages [18,19]. Therefore, early and regular physical activity could be a key factor for preventing long-term health deterioration and cognitive decline. Light physical activity is recommended as part of everyday living activities for people with DS to maintain a healthy weight, particularly when barriers restrict the pursuit of more-vigorous physical activity [20]. Furthermore, the observed association between the sedentary lifestyle of parents and the physical activity of their adolescents with DS suggests that interventions might be more effective when addressed at a family level [21]. Unlike in the general population, where individual motivation plays a primary role, individuals with DS often depend on family or caregivers to facilitate physical activity. Given that physically active parents are more likely to have active children, promoting physical activity behaviors among parents and siblings may be a beneficial strategy [22]. Encouraging families and peers to engage in active lifestyles may increase participation and adherence.

## 4. Discussion

**Figure 1:**
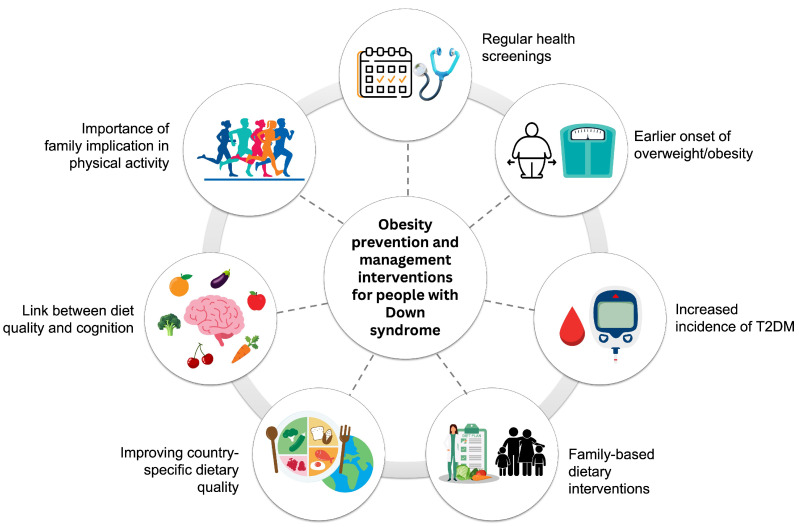
Summary of the key points that must be taken into consideration for the prevention and management interventions of overweight and obesity in people with Down syndrome.

Our findings within the GO-DS21 project, together with other previously published guidelines, shed light on critical health aspects in DS, revealing several key insights for the prevention of overweight and obesity (Figure 1). By integrating our primary data with existing evidence, we establish actionable recommendations that address the early onset of comorbidities—such as type 2 diabetes—in individuals with DS. Furthermore, we highlight the significant influence of family environment (including parental BMI) and lifestyle, specifically the importance of diet quality and physical activity for both metabolic and cognitive health. The following points summarize these clinical recommendations:

- Co-occurring conditions are highly prevalent in individuals with DS and appear earlier in life; therefore, we recommend regular health screenings and personalized interventions from early stages of development.
- Individuals with DS, although presenting a similar prevalence of obesity as the general population, are more prone to earlier onset of overweight or obesity, requiring proactive weight monitoring from childhood through adulthood.
- A strong link between obesity and an increased incidence of type 2 diabetes mellitus is observed in people with DS at younger ages, highlighting the need for early metabolic assessments.
- Parental BMI has a significant influence on the BMI of individuals with DS; consequently, clinical strategies should prioritize family-based dietary interventions. Moreover, a high-quality diet during maternal pregnancy must be highlighted as a preventive measure.
- The overall dietary quality in people with DS can be improved, considering that country-specific dietary advice is essential due to the different dietary qualities observed across European countries.
- Better cognitive function could be linked to a higher dietary quality, which is crucial in a neurodevelopmental disorder such as DS.
- Exercise is directly correlated with weight control and management. Clinicians should encourage the family and peers of individuals with DS to engage in active lifestyles to support their participation and adherence to physical activities.

These guidelines are derived primarily from observational studies; therefore, causal inferences should be made with caution. The absence of intervention data may limit the strength of some conclusions.

## 5. Conclusions

The findings discussed should be considered to prevent or effectively treat overweight or obesity in people with DS, whilst considering the associated comorbidities and individual features that characterize this condition. This approach must extend beyond simple dietary adjustments, incorporating adapted physical exercise tailored to specific needs, abilities and environment. Most critically, success will depend on the active and consistent involvement of families and peers for the maintenance of healthy habits and ensuring meaningful, sustainable improvements.

## Figures and Tables

**Table 1:** Overall characteristics of the study population analyzed in the GO-DS21 study (*n* = 185).

Characteristic	Overall (*n* = 185)
**Age, mean (SD); range**	24.7 (9.4); (12–45 years)
**Sex, ** ***n *** **(%)**	
Female	83 (44.9%)
Male	102 (55.1%)
**BMI, mean (SD); range**	26.5 (6.2); (16.48–48.32)
**Weight category, ** ***n *** **(%)**	
Underweight	7 (3.8%)
Normal weight	87 (47.0%)
Overweight	47 (25.4%)
Obese	44 (23.8%)

## Data Availability

In this brief communication, a series of recommendations for the management and treatment of overweight and obesity in people with Down syndrome are presented, so there is no data to be made available to the scientific community.

## Abbreviations

The following abbreviations are used in this manuscript:

DS	Down syndrome
GO-DS21	Gene overdosage and comorbidities during the early lifetime in Down syndrome
BMI	Body mass index
IQ	Intelligence quotient
